# 2-(1*H*-Benzotriazol-1-yl)-1-(2-fluoro­benzo­yl)ethyl 4-methyl­benzoate

**DOI:** 10.1107/S1600536809029481

**Published:** 2009-07-29

**Authors:** Wu-Lan Zeng, Fang-Fang Jian

**Affiliations:** a Microscale Science Institute, Department of Chemistry and Chemical Engineering, Weifang University, Weifang 261061, People’s Republic of China

## Abstract

In the crystal structure of the title compound, C_23_H_18_FN_3_O_3_, inter­molecular C—H⋯N hydrogen bonds link the mol­ecules into chains extended along the *c* axis. The packing is further stabilized by weak C—H⋯O and C—H⋯F inter­actions. The F atom is disordered over two equally occupied 1- and 5-positions of the benzene ring.

## Related literature

For the pharmacological activity of 1*H*-benzotriazole and its derivatives, see: Chen & Wu (2005 ▶). For bond-length data, see: Allen *et al.* (1987 ▶).
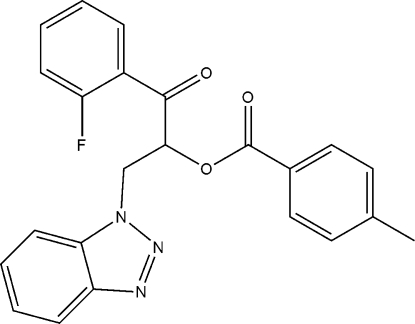


## Experimental

### 

#### Crystal data

C_23_H_18_FN_3_O_3_
*M* *_r_* = 403.40
Monoclinic, 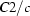

*a* = 20.478 (4) Å
*b* = 19.570 (4) Å
*c* = 9.969 (2) Å
β = 107.12 (3)°
*V* = 3818.0 (13) Å^3^
*Z* = 8
Mo *K*α radiation
μ = 0.10 mm^−1^
*T* = 293 K
0.10 × 0.06 × 0.02 mm

#### Data collection

Bruker SMART CCD diffractometer
Absorption correction: multi-scan (*SADABS*; Bruker, 1997 ▶) *T*_min_ = 0.990, *T*_max_ = 0.998
19476 measured reflections
3368 independent reflections
2698 reflections with *I* > 2σ(*I*)
*R*_int_ = 0.058

#### Refinement

*R*[*F* ^2^ > 2σ(*F* ^2^)] = 0.064
*wR*(*F* ^2^) = 0.177
*S* = 1.08
3368 reflections
282 parameters
2 restraints
H-atom parameters constrained
Δρ_max_ = 0.38 e Å^−3^
Δρ_min_ = −0.27 e Å^−3^

### 

Data collection: *SMART* (Bruker, 1997 ▶); cell refinement: *SAINT* (Bruker, 1997 ▶); data reduction: *SAINT*; program(s) used to solve structure: *SHELXS97* (Sheldrick, 2008 ▶); program(s) used to refine structure: *SHELXL97* (Sheldrick, 2008 ▶); molecular graphics: *SHELXTL* (Sheldrick, 2008 ▶); software used to prepare material for publication: *SHELXTL*.

## Supplementary Material

Crystal structure: contains datablocks global, I. DOI: 10.1107/S1600536809029481/at2823sup1.cif

Structure factors: contains datablocks I. DOI: 10.1107/S1600536809029481/at2823Isup2.hkl

Additional supplementary materials:  crystallographic information; 3D view; checkCIF report

## Figures and Tables

**Table 1:** Hydrogen-bond geometry (Å, °)

*D*—H⋯*A*	*D*—H	H⋯*A*	*D*⋯*A*	*D*—H⋯*A*
C8—H8⋯F1	0.98	2.24	2.938 (5)	127
C9—H9*A*⋯N2^i^	0.97	2.55	3.515 (3)	174
C12—H12⋯O1^ii^	0.93	2.48	3.118 (4)	126
C22—H22⋯O3^iii^	0.93	2.48	3.259 (4)	142
C23—H23*C*⋯F1′^iv^	0.96	2.37	3.090 (5)	131

Symmetry codes: (i) 
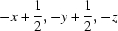
; (ii) 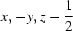; (iii) 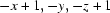; (iv) 
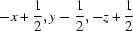
.

**Fig. 1.**
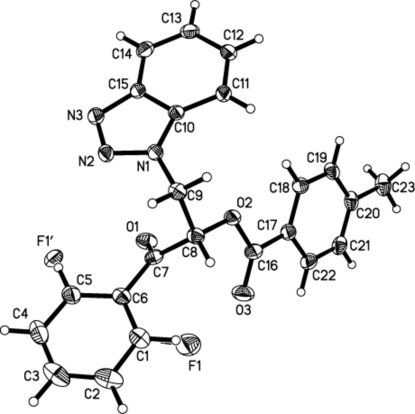
The molecular structure of (I), drawn with 30% probability ellipsoids.

## supplementary crystallographic information

### Comment

1*H*-Benzotriazoles and its derivatives are an important class of compounds because they exhibit a broad spectrum of pharmacological activities such as antifungal, antitumor and antineoplastic activities (Chen & Wu, 2005). We report here the synthesis and structure of the title compound, (I) (Fig. 1), as part of our ongoing studies on new benzotriazole compounds with higher bioactivity.

All the bond lengths (Allen *et al.*, 1987) and angles in (I) are within their normal ranges. The dihedral angle between the triazole ring (N1–N3/C10/C15) and the benzene ring (C10–C15) is 2.86 (12)°. The dihedral angles between the triazole ring and the C1–C6 and C17–C22 aromatic rings are 4.78 (13)° and 65.34 (13)°, respectively. The dihedral angle between the C1—C6 and C17–C22 rings is 62.04 (14)°. Molecule (I) is chiral. In the crystal structure, intermolecular C—H···N hydrogen bonds (Table1) link the molecules into chains extended along the c axis. The packing (Fig. 2) is further stabilized by weak C—H···O and C—H···F interactions (Table 1).

### Experimental

Bromine (3.2 g, 0.02 mol) was added dropwise to a solution of 3-(1*H*-benzo[*d*][1,2,3]triazol-1-yl)-1-(2-fluorophenyl)propan-1-one (5.38 g, 0.02 mol) and sodium acetate (1.6 g, 0.02 mol) in acetic acid (50 ml). The reaction proceeded for 7 h. Water (50 ml) and chloroform (20 ml) were then added. The organic layer was washed successively with saturated sodium bicarbonate solution and brine, dried over anhydrous magnesium sulfate and the chloroform solution filtered. It was cooled with ice-water, and then an acetone solution (10 ml) of 4-methylbenzoic acid (2.72 g, 0.02 mol) and triethylamine (2.8 ml) was added. The mixture was stirred with ice-water for 6 h. The solution was then filtered and concentrated. Single crystals of (I) were obtained by slow evaporation of an acetone-ethylacetate (3:1 *v*/*v*) solution at room temperature over a period of one week.

### Refinement

All H atoms were located in difference Fourier maps and constrained to ride on their parent atoms, with C—H distances in the range 0.93–0.97 Å, and with *U*_iso_(H) = 1.2 *U*_eq_(C) and 1.5 *U*_eq_(methyl C) H atoms. The F atom is disordered over two equally occupied positions on the 1 and 5-positions of the benzene ring.

### Crystal data

**Table:**

C_23_H_18_FN_3_O_3_	*F*(000) = 1680
*M**_r_* = 403.40	*D*_x_ = 1.404 Mg m^−^^3^
Monoclinic, *C*2/*c*	Mo *K*α radiation, λ = 0.71073 Å
Hall symbol: -C 2yc	Cell parameters from 3992 reflections
*a* = 20.478 (4) Å	θ = 2.1–25.0°
*b* = 19.570 (4) Å	µ = 0.10 mm^−^^1^
*c* = 9.969 (2) Å	*T* = 293 K
β = 107.12 (3)°	Block, colourless
*V* = 3818.0 (13) Å^3^	0.10 × 0.06 × 0.02 mm
*Z* = 8	

### Data collection

**Table:**

Bruker SMART CCD diffractometer	3368 independent reflections
Radiation source: fine-focus sealed tube	2698 reflections with *I* > 2σ(*I*)
graphite	*R*_int_ = 0.058
ω scans	θ_max_ = 25.0°, θ_min_ = 2.1°
Absorption correction: multi-scan (*SADABS*; Bruker, 1997)	*h* = −24→24
*T*_min_ = 0.990, *T*_max_ = 0.998	*k* = −23→23
19476 measured reflections	*l* = −11→11

### Refinement

**Table:**

Refinement on *F*^2^	Secondary atom site location: difference Fourier map
Least-squares matrix: full	Hydrogen site location: inferred from neighbouring sites
*R*[*F*^2^ > 2σ(*F*^2^)] = 0.064	H-atom parameters constrained
*wR*(*F*^2^) = 0.177	*w* = 1/[σ^2^(*F*_o_^2^) + (0.0959*P*)^2^ + 1.3136*P*] where *P* = (*F*_o_^2^ + 2*F*_c_^2^)/3
*S* = 1.08	(Δ/σ)_max_ = 0.031
3368 reflections	Δρ_max_ = 0.38 e Å^−^^3^
282 parameters	Δρ_min_ = −0.26 e Å^−^^3^
2 restraints	Extinction correction: *SHELXL97* (Sheldrick, 2008), Fc^*^=kFc[1+0.001xFc^2^λ^3^/sin(2θ)]^-1/4^
Primary atom site location: structure-invariant direct methods	Extinction coefficient: 0.0028 (6)

### Special details

**Table:**

Geometry. All e.s.d.'s (except the e.s.d. in the dihedral angle between two l.s. planes) are estimated using the full covariance matrix. The cell e.s.d.'s are taken into account individually in the estimation of e.s.d.'s in distances, angles and torsion angles; correlations between e.s.d.'s in cell parameters are only used when they are defined by crystal symmetry. An approximate (isotropic) treatment of cell e.s.d.'s is used for estimating e.s.d.'s involving l.s. planes.
Refinement. Refinement of *F*^2^ against ALL reflections. The weighted *R*-factor *wR* and goodness of fit *S* are based on *F*^2^, conventional *R*-factors *R* are based on *F*, with *F* set to zero for negative *F*^2^. The threshold expression of *F*^2^ > σ(*F*^2^) is used only for calculating *R*-factors(gt) *etc*. and is not relevant to the choice of reflections for refinement. *R*-factors based on *F*^2^ are statistically about twice as large as those based on *F*, and *R*- factors based on ALL data will be even larger.

### Fractional atomic coordinates and isotropic or equivalent isotropic displacement parameters (Å^2^)

**Table:**

	*x*	*y*	*z*	*U*_iso_*/*U*_eq_	Occ. (<1)
F1	0.46661 (19)	0.21126 (19)	0.3054 (4)	0.0709 (10)	0.50
F1'	0.24580 (17)	0.2535 (2)	0.3171 (4)	0.0717 (12)	0.50
O1	0.29447 (10)	0.12509 (10)	0.30001 (18)	0.0462 (5)	
O2	0.35449 (9)	0.05690 (8)	0.13268 (16)	0.0371 (5)	
O3	0.43939 (11)	0.06667 (10)	0.3332 (2)	0.0622 (7)	
N1	0.23322 (11)	0.12861 (10)	−0.02778 (19)	0.0326 (5)	
N2	0.19010 (12)	0.17037 (10)	0.0132 (2)	0.0378 (5)	
N3	0.13058 (12)	0.14092 (11)	−0.0135 (2)	0.0401 (6)	
C1	0.41929 (16)	0.25100 (14)	0.3243 (3)	0.0452 (7)	
H1A	0.4518	0.2210	0.3024	0.054*	0.50
C2	0.44099 (19)	0.31500 (17)	0.3776 (3)	0.0602 (9)	
H2	0.4862	0.3286	0.3936	0.072*	
C3	0.3935 (2)	0.35812 (16)	0.4063 (3)	0.0649 (10)	
H3	0.4064	0.4018	0.4405	0.078*	
C4	0.3281 (2)	0.33705 (15)	0.3850 (3)	0.0603 (9)	
H4	0.2964	0.3662	0.4053	0.072*	
C5	0.30868 (16)	0.27283 (14)	0.3337 (3)	0.0487 (8)	
H5A	0.2624	0.2585	0.3204	0.058*	0.50
C6	0.35371 (14)	0.22830 (13)	0.3012 (2)	0.0367 (6)	
C7	0.33146 (13)	0.15790 (13)	0.2505 (2)	0.0349 (6)	
C8	0.35328 (14)	0.12962 (12)	0.1289 (2)	0.0358 (6)	
H8	0.3989	0.1469	0.1339	0.043*	
C9	0.30282 (13)	0.15017 (12)	−0.0110 (2)	0.0365 (6)	
H9A	0.3036	0.1995	−0.0199	0.044*	
H9B	0.3178	0.1307	−0.0864	0.044*	
C10	0.20015 (13)	0.07007 (12)	−0.0843 (2)	0.0312 (6)	
C11	0.21918 (14)	0.01290 (12)	−0.1472 (2)	0.0361 (6)	
H11	0.2632	0.0075	−0.1538	0.043*	
C12	0.16911 (14)	−0.03490 (13)	−0.1989 (3)	0.0397 (7)	
H12	0.1795	−0.0740	−0.2417	0.048*	
C13	0.10262 (14)	−0.02655 (14)	−0.1889 (3)	0.0435 (7)	
H13	0.0701	−0.0600	−0.2261	0.052*	
C14	0.08430 (14)	0.02928 (14)	−0.1262 (3)	0.0418 (7)	
H14	0.0403	0.0342	−0.1187	0.050*	
C15	0.13458 (13)	0.07866 (12)	−0.0740 (2)	0.0341 (6)	
C16	0.40188 (14)	0.03020 (14)	0.2459 (3)	0.0439 (7)	
C17	0.40226 (13)	−0.04463 (13)	0.2469 (2)	0.0382 (6)	
C18	0.36063 (14)	−0.08304 (13)	0.1382 (3)	0.0391 (6)	
H18	0.3305	−0.0613	0.0618	0.047*	
C19	0.36382 (15)	−0.15284 (13)	0.1430 (3)	0.0397 (6)	
H19	0.3358	−0.1781	0.0692	0.048*	
C20	0.40830 (14)	−0.18695 (14)	0.2562 (3)	0.0418 (7)	
C21	0.44946 (15)	−0.14802 (15)	0.3646 (3)	0.0448 (7)	
H21	0.4795	−0.1697	0.4412	0.054*	
C22	0.44658 (14)	−0.07813 (14)	0.3607 (3)	0.0449 (7)	
H22	0.4745	−0.0529	0.4346	0.054*	
C23	0.41090 (17)	−0.26339 (14)	0.2594 (3)	0.0553 (8)	
H23A	0.4499	−0.2781	0.3338	0.083*	
H23B	0.4145	−0.2801	0.1714	0.083*	
H23C	0.3700	−0.2809	0.2751	0.083*	

### Atomic displacement parameters (Å^2^)

**Table:**

	*U*^11^	*U*^22^	*U*^33^	*U*^12^	*U*^13^	*U*^23^
F1	0.056 (2)	0.074 (2)	0.086 (3)	−0.018 (2)	0.027 (2)	−0.013 (2)
F1'	0.034 (2)	0.099 (3)	0.070 (2)	0.0195 (19)	−0.0037 (17)	−0.037 (2)
O1	0.0466 (12)	0.0547 (12)	0.0385 (10)	−0.0151 (9)	0.0143 (9)	−0.0042 (9)
O2	0.0374 (10)	0.0336 (9)	0.0341 (9)	0.0032 (8)	0.0012 (8)	−0.0012 (7)
O3	0.0624 (15)	0.0522 (12)	0.0512 (13)	0.0025 (11)	−0.0156 (11)	−0.0096 (10)
N1	0.0364 (12)	0.0303 (11)	0.0287 (11)	0.0038 (9)	0.0059 (9)	0.0007 (8)
N2	0.0453 (14)	0.0351 (11)	0.0301 (11)	0.0105 (10)	0.0065 (10)	−0.0004 (9)
N3	0.0426 (14)	0.0419 (13)	0.0333 (12)	0.0087 (10)	0.0069 (10)	−0.0014 (9)
C1	0.0571 (19)	0.0436 (16)	0.0391 (15)	−0.0061 (14)	0.0209 (14)	0.0011 (12)
C2	0.078 (2)	0.060 (2)	0.0430 (17)	−0.0303 (18)	0.0181 (16)	0.0019 (14)
C3	0.113 (3)	0.0395 (17)	0.0376 (17)	−0.0130 (19)	0.0156 (19)	0.0022 (13)
C4	0.089 (3)	0.0447 (17)	0.0426 (17)	0.0191 (18)	0.0129 (17)	−0.0022 (14)
C5	0.057 (2)	0.0470 (17)	0.0339 (14)	0.0092 (14)	0.0009 (13)	−0.0034 (12)
C6	0.0462 (17)	0.0371 (14)	0.0244 (12)	−0.0004 (12)	0.0069 (11)	0.0021 (10)
C7	0.0308 (14)	0.0422 (14)	0.0285 (12)	−0.0025 (11)	0.0039 (11)	0.0057 (11)
C8	0.0369 (15)	0.0328 (13)	0.0361 (14)	−0.0027 (11)	0.0082 (12)	−0.0007 (11)
C9	0.0429 (16)	0.0329 (13)	0.0321 (13)	−0.0008 (11)	0.0082 (12)	0.0018 (10)
C10	0.0348 (14)	0.0309 (13)	0.0245 (12)	0.0034 (10)	0.0034 (10)	0.0022 (10)
C11	0.0377 (15)	0.0378 (14)	0.0314 (13)	0.0071 (11)	0.0079 (11)	−0.0006 (11)
C12	0.0440 (17)	0.0340 (14)	0.0365 (14)	0.0032 (11)	0.0046 (12)	−0.0061 (11)
C13	0.0387 (16)	0.0416 (15)	0.0413 (15)	−0.0038 (12)	−0.0020 (12)	−0.0023 (12)
C14	0.0334 (15)	0.0512 (17)	0.0378 (14)	0.0042 (12)	0.0058 (12)	0.0023 (12)
C15	0.0370 (15)	0.0344 (13)	0.0268 (12)	0.0075 (11)	0.0029 (11)	0.0016 (10)
C16	0.0405 (16)	0.0475 (16)	0.0356 (15)	0.0056 (13)	−0.0012 (13)	−0.0016 (12)
C17	0.0388 (15)	0.0419 (15)	0.0310 (13)	0.0085 (12)	0.0058 (11)	−0.0004 (11)
C18	0.0425 (16)	0.0430 (15)	0.0285 (13)	0.0082 (12)	0.0055 (12)	0.0028 (11)
C19	0.0514 (17)	0.0407 (15)	0.0268 (13)	0.0093 (12)	0.0110 (12)	−0.0001 (11)
C20	0.0509 (18)	0.0456 (16)	0.0364 (14)	0.0138 (13)	0.0244 (13)	0.0071 (12)
C21	0.0438 (17)	0.0564 (18)	0.0339 (14)	0.0192 (14)	0.0109 (13)	0.0098 (13)
C22	0.0388 (16)	0.0559 (18)	0.0347 (14)	0.0100 (13)	0.0025 (12)	0.0005 (12)
C23	0.072 (2)	0.0493 (17)	0.0518 (17)	0.0197 (15)	0.0292 (16)	0.0130 (14)

### Geometric parameters (Å, °)

**Table:**

F1—C1	1.298 (4)	C9—H9A	0.9700
F1—H1A	0.3515	C9—H9B	0.9700
F1'—C5	1.305 (4)	C10—C15	1.387 (3)
F1'—H5A	0.3449	C10—C11	1.393 (3)
O1—C7	1.204 (3)	C11—C12	1.371 (4)
O2—C16	1.358 (3)	C11—H11	0.9300
O2—C8	1.424 (3)	C12—C13	1.403 (4)
O3—C16	1.209 (3)	C12—H12	0.9300
N1—N2	1.352 (3)	C13—C14	1.365 (4)
N1—C10	1.365 (3)	C13—H13	0.9300
N1—C9	1.448 (3)	C14—C15	1.396 (4)
N2—N3	1.303 (3)	C14—H14	0.9300
N3—C15	1.373 (3)	C16—C17	1.465 (4)
C1—C6	1.368 (4)	C17—C18	1.387 (4)
C1—C2	1.382 (4)	C17—C22	1.390 (4)
C1—H1A	0.9599	C18—C19	1.368 (3)
C2—C3	1.379 (5)	C18—H18	0.9300
C2—H2	0.9300	C19—C20	1.394 (4)
C3—C4	1.357 (5)	C19—H19	0.9300
C3—H3	0.9300	C20—C21	1.386 (4)
C4—C5	1.371 (4)	C20—C23	1.497 (4)
C4—H4	0.9300	C21—C22	1.369 (4)
C5—C6	1.375 (4)	C21—H21	0.9300
C5—H5A	0.9601	C22—H22	0.9300
C6—C7	1.492 (4)	C23—H23A	0.9600
C7—C8	1.515 (3)	C23—H23B	0.9600
C8—C9	1.525 (3)	C23—H23C	0.9600
C8—H8	0.9800		
			
C1—F1—H1A	13.4	H9A—C9—H9B	107.6
C5—F1'—H5A	1.9	N1—C10—C15	104.0 (2)
C16—O2—C8	114.16 (19)	N1—C10—C11	133.5 (2)
N2—N1—C10	110.1 (2)	C15—C10—C11	122.4 (2)
N2—N1—C9	119.8 (2)	C12—C11—C10	116.1 (2)
C10—N1—C9	130.1 (2)	C12—C11—H11	122.0
N3—N2—N1	109.03 (19)	C10—C11—H11	122.0
N2—N3—C15	108.0 (2)	C11—C12—C13	121.9 (2)
F1—C1—C6	121.2 (3)	C11—C12—H12	119.1
F1—C1—C2	115.4 (3)	C13—C12—H12	119.1
C6—C1—C2	123.2 (3)	C14—C13—C12	121.9 (3)
F1—C1—H1A	4.9	C14—C13—H13	119.0
C6—C1—H1A	118.4	C12—C13—H13	119.0
C2—C1—H1A	118.4	C13—C14—C15	116.9 (3)
C3—C2—C1	117.9 (3)	C13—C14—H14	121.5
C3—C2—H2	121.1	C15—C14—H14	121.5
C1—C2—H2	121.1	N3—C15—C10	108.9 (2)
C4—C3—C2	120.3 (3)	N3—C15—C14	130.2 (2)
C4—C3—H3	119.8	C10—C15—C14	120.8 (2)
C2—C3—H3	119.8	O3—C16—O2	121.2 (2)
C3—C4—C5	120.1 (3)	O3—C16—C17	125.7 (2)
C3—C4—H4	119.9	O2—C16—C17	113.1 (2)
C5—C4—H4	119.9	C18—C17—C22	119.0 (2)
F1'—C5—C4	118.7 (3)	C18—C17—C16	122.4 (2)
F1'—C5—C6	119.5 (3)	C22—C17—C16	118.6 (2)
C4—C5—C6	121.8 (3)	C19—C18—C17	120.1 (2)
F1'—C5—H5A	0.7	C19—C18—H18	119.9
C4—C5—H5A	119.1	C17—C18—H18	119.9
C6—C5—H5A	119.1	C18—C19—C20	121.3 (2)
C1—C6—C5	116.6 (3)	C18—C19—H19	119.3
C1—C6—C7	122.9 (2)	C20—C19—H19	119.4
C5—C6—C7	120.4 (3)	C21—C20—C19	118.0 (2)
O1—C7—C6	121.3 (2)	C21—C20—C23	121.5 (3)
O1—C7—C8	120.2 (2)	C19—C20—C23	120.4 (3)
C6—C7—C8	118.4 (2)	C22—C21—C20	121.0 (2)
O2—C8—C7	110.6 (2)	C22—C21—H21	119.5
O2—C8—C9	106.83 (19)	C20—C21—H21	119.5
C7—C8—C9	110.8 (2)	C21—C22—C17	120.4 (3)
O2—C8—H8	109.5	C21—C22—H22	119.8
C7—C8—H8	109.5	C17—C22—H22	119.8
C9—C8—H8	109.5	C20—C23—H23A	109.5
N1—C9—C8	114.0 (2)	C20—C23—H23B	109.5
N1—C9—H9A	108.7	H23A—C23—H23B	109.5
C8—C9—H9A	108.7	C20—C23—H23C	109.5
N1—C9—H9B	108.7	H23A—C23—H23C	109.5
C8—C9—H9B	108.7	H23B—C23—H23C	109.5
			
C10—N1—N2—N3	0.5 (2)	C9—N1—C10—C15	−178.9 (2)
C9—N1—N2—N3	179.16 (19)	N2—N1—C10—C11	176.5 (2)
N1—N2—N3—C15	−0.3 (2)	C9—N1—C10—C11	−2.0 (4)
F1—C1—C2—C3	−176.8 (3)	N1—C10—C11—C12	−176.5 (2)
C6—C1—C2—C3	−1.2 (4)	C15—C10—C11—C12	−0.1 (3)
C1—C2—C3—C4	1.3 (4)	C10—C11—C12—C13	0.1 (4)
C2—C3—C4—C5	−0.5 (4)	C11—C12—C13—C14	−0.6 (4)
C3—C4—C5—F1'	178.6 (3)	C12—C13—C14—C15	1.0 (4)
C3—C4—C5—C6	−0.6 (4)	N2—N3—C15—C10	0.0 (3)
F1—C1—C6—C5	175.6 (3)	N2—N3—C15—C14	−177.3 (2)
C2—C1—C6—C5	0.1 (4)	N1—C10—C15—N3	0.2 (2)
F1—C1—C6—C7	−1.4 (4)	C11—C10—C15—N3	−177.1 (2)
C2—C1—C6—C7	−176.8 (2)	N1—C10—C15—C14	177.9 (2)
F1'—C5—C6—C1	−178.4 (3)	C11—C10—C15—C14	0.5 (4)
C4—C5—C6—C1	0.8 (4)	C13—C14—C15—N3	176.1 (2)
F1'—C5—C6—C7	−1.4 (4)	C13—C14—C15—C10	−1.0 (4)
C4—C5—C6—C7	177.8 (2)	C8—O2—C16—O3	−0.7 (4)
C1—C6—C7—O1	137.2 (3)	C8—O2—C16—C17	−179.7 (2)
C5—C6—C7—O1	−39.6 (4)	O3—C16—C17—C18	−175.6 (3)
C1—C6—C7—C8	−46.1 (3)	O2—C16—C17—C18	3.4 (4)
C5—C6—C7—C8	137.1 (2)	O3—C16—C17—C22	3.7 (4)
C16—O2—C8—C7	−65.0 (3)	O2—C16—C17—C22	−177.3 (2)
C16—O2—C8—C9	174.4 (2)	C22—C17—C18—C19	−0.5 (4)
O1—C7—C8—O2	−28.4 (3)	C16—C17—C18—C19	178.8 (2)
C6—C7—C8—O2	154.9 (2)	C17—C18—C19—C20	0.2 (4)
O1—C7—C8—C9	89.9 (3)	C18—C19—C20—C21	0.0 (4)
C6—C7—C8—C9	−86.8 (3)	C18—C19—C20—C23	179.9 (3)
N2—N1—C9—C8	90.5 (3)	C19—C20—C21—C22	0.0 (4)
C10—N1—C9—C8	−91.1 (3)	C23—C20—C21—C22	−179.9 (3)
O2—C8—C9—N1	63.0 (3)	C20—C21—C22—C17	−0.3 (4)
C7—C8—C9—N1	−57.5 (3)	C18—C17—C22—C21	0.5 (4)
N2—N1—C10—C15	−0.4 (2)	C16—C17—C22—C21	−178.8 (2)

### Hydrogen-bond geometry (Å, °)

**Table:**

*D*—H···*A*	*D*—H	H···*A*	*D*···*A*	*D*—H···*A*
C8—H8···F1	0.98	2.24	2.938 (5)	127
C9—H9A···N2^i^	0.97	2.55	3.515 (3)	174
C12—H12···O1^ii^	0.93	2.48	3.118 (4)	126
C18—H18···O2	0.93	2.43	2.741 (3)	100
C22—H22···O3^iii^	0.93	2.48	3.259 (4)	142
C23—H23C···F1'^iv^	0.96	2.37	3.090 (5)	131

Symmetry codes: (i) −*x*+1/2, −*y*+1/2, −*z*; (ii) *x*, −*y*, *z*−1/2; (iii) −*x*+1, −*y*, −*z*+1; (iv) −*x*+1/2, *y*−1/2, −*z*+1/2.
